# The effect of CD14 and TLR4 gene polimorphisms on asthma phenotypes in adult Turkish asthma patients: a genetic study

**DOI:** 10.1186/1471-2466-14-20

**Published:** 2014-02-13

**Authors:** Füsun Şahin, Pınar Yıldız, Ayşegül Kuskucu, Mert Ahmet Kuskucu, Nilgün Karaca, Kenan Midilli

**Affiliations:** 1 Department of Chest Diseases, Yedikule Chest Diseases and Surgery Training and Research Hospital, Zeytinburnu/İstanbul, 34760, Turkey; 2 Department of Medical Genetics, Yeditepe University School of Medicine, İstanbul, Turkey; 3 Department of Medical Microbiology, Cerrahpaşa School of Medicine, İstanbul University, İstanbul, Turkey; 4 Department of Biotechnology, Yeditepe Univesity Institute of Science, İstanbul, Turkey

**Keywords:** Asthma, Atopy, Gen polymorphism, CD14, TLR4, Total IgE, Eosinophil

## Abstract

**Background:**

Endotoxins stimulate T helper 1 cell maturation and send a negative signal to T helper 2 polarisation. This causes a decrease IgE levels and prevents atopy (Hygiene hypothesis). It is shown that this response is under genetic control by polymorphisms in CD14 and TLR4 genes in some researchs. We aimed to investigate the effects of genetic variants of CD14 (−) and TLR4 (Asp299Gly, Thr399Ile) genes on asthma phenotypes in adults with asthma.

**Methods:**

Asthma patients (n = 131) and healthy control cases (n = 75) were included in the study. Relations between CD14 C-159 T, TLR4 299 and TLR4 399 genotypes and duration of asthma history of allergic rhinitis-dermatitis, total IgE, eosinophil, skin prick test, forced expiratory volume 1 (FEV1) and severity of disease were evaluated. Real time PCR (RT-PCR) was used for genotyping.

**Results:**

For CD14-159, presence of the C allele (CC + CT) was more frequent among those with low median log (logarithm) IgE levels, but no statistically significant difference in all asthma group (p = 0.09). C allele was significantly correlated with low total IgE levels and T allele with high total IgE levels in atopics (p = 0.04). CC + CT genotype was more frequent in moderate and severe asthma group in atopics (p = 0.049). TLR4 299 and TLR4 399 genotypes and asthma phenotypes were not found to be significantly correlated (p > 0.05).

**Conclusions:**

Total IgE levels were found to be low among patients with the CC + CT genotype, and high among patients with the TT genotype contrary to the results of many other studies, which is therefore an important finding. Another important finding was that the C allele is a risk factor for moderate and severe asthma.

## Background

Asthma is a complex disease in which pathogenesis is related to both genetic and environmental factors [1]. In 1989, Strachan [2] put “hygiene hypothesis”, which proposed that reduced exposure to infectious microorganisms in childhood leads to an increased risk of allergic disease in later years. It is thought that bacteria stimulate T helper 1 (Th1) maturation, inhibite T helper 2 (Th2) polarisation and ultimately decrease IgE levels. Innate immunity utilizes the Toll-like receptor (TLR) family to identify pathogens. TLRs are expressed on the cell surface and initiate immune defence by recognising structures specific to microorganisms, such as lipopolysaccharide (LPS) [3]. Pathogens also contain complexes called “pathogen associated molecular patterns (PAMPs)”, which are not present in the host [4]. During an infection, macrophages recognise PAMPs via TLRs. Dendritic cell activation occurs due to the stimulation of TLRs, enabling communication between peripheral and lymphatic tissues; in turn T cells are activated, which is the most important component of the acquired immune system [5,6]. Numerous studies have implicated TLR2, TLR4 and TLR9 in the pathogenesis of asthma or atopy [7-9]. CD14 is a multifunctional receptor on macrophages and monocytes that binds endotoxin and other bacterial wall components. CD14 promotes immune activation by facilitating the presentation of LPS to TLR4 [10], which triggers interleukin-(IL)-12 and IL-18 signalling and interferon-gamma production. As a result a shift to Th1 occurs and atopy is prevented. In recent years it was shown that this response is under genetic control and is specifically determined by polymorphisms in the CD14. The TLR4 single nucleotide polymorphisms A896G and C1196T (result in the amino acid substitutions Asp299Gly → RefSNP ID: rs4986790 and Thr399Ile → RefSNP ID: rs4986791, respectively) and affect the extracellular domain of the TLR4 receptor [7,11]. These two polymorphisms modify the receptor’s response to endotoxin, which is an important trigger of asthma [7]. Similarly, the CD14 polymorphism C-159 T (RefSNP ID: rs2569190) has been shown to influence serum IgE levels and skin prick test responses to allergens [7,12]. Allergic disease may emerge through exposure to endotoxins, although responsiveness may differ according to genetic factors [11,12]. In this way the effect of endotoxins may vary according to the atopic condition of the host. As a result it is thought that the effect of the CD14 -159 genotype (CC, TT or CT) on the asthma phenotype (in terms of total IgE levels) may also vary and numerous studies have been conducted to investigate the relationship between CD14 variants and total IgE levels [13]. In the present study, we investigated the effects of genetic variants of the CD14(C-159 T) and TLR4 (Asp299Gly and Thr399Ile) polymorphisms on adult asthma phenotypes specifically the severity of disease, total IgE concentrations and eosinophil counts. This study is important because it is the first to evaluate together with the effects of both CD14 and TLR4 genetic variants on disease in adult patients with asthma in Turkey.

## Methods

### Study subjects

Patients with asthma (n = 131) and healthy controls (n = 75) who presented to the Third Chest Diseases Outpatient Clinic at the Yedikule Chest Diseases and Surgery Training and Research Hospital between January 2010 and December 2011 were included in this study. A power analysis was performed for each gene polymorphism (CD14 -159, TLR4 299, TLR4 399) at the beginning of the study (to determine the necessary sample size). We determined the allele frequencies of all polymorphisms (CD14 -159, TLR4 299, TLR4 399) according to the results of the studies from our region [13,14] and a meta-analysis (that included European, East Asian and Indian populations) [15]. The allele frequency of CD14 C-159 T was approximately 75% in the asthma group, 53% in the control group. The power analysis was performed according to these values. For the C-159 T polymorphism, the required sample size was 74 for each group (Approximate Test). The allele frequencies for TLR4 299 and TLR4 399 were approximately 20% in the asthma group, 5% in the control group, an again the reguired sample size was determined to be 70 for each group (Approximate Test). The number of patients in our study met these criteria.

Asthma was diagnosed according to the Global Initiative for Asthma (GINA) guidelines in patients with recurrent dyspnoea, wheezing attacks and positive airway reversibility test (12% or > 200 mL increase in FEV1 after 400 μg salbutamol inhalation) [16]. Patients who had an attack in the previous month and/or were treated with systemic steroids, took specific allergen immunotherapy, had a personal or family history of tuberculosis, or had active pathology or sequelae upon chest X-ray examination were excluded. The control group was composed of healthy individuals with no personal or family history of atopy or tuberculosis. Ninety six patients with allergic symptoms or a history of allergy and who showed a positive reaction to at least one allergen on skin prick testing were classified as having atopic asthma, and 35 patients with no allergic symptoms, no personal history and negative skin test results were classified as having non-atopic asthma. This study was performed in accordance with the principles of the Declaration of Helsinki (2008) and approved by the ethics committee of Yeditepe University. Written and signed informed consent was obtained from all participants.

### Measurements

Spirometric evaluations were performed with a MasterScope-PC (Jaeger, Germany), total IgE levels were measured with a Unicel D × I 800 analyser (Beckman Coulter, (US) and eosinophil counts were determined using an ABX Pentra 120 system (Impedance&Optical, Minnesota, USA). All patients with asthma underwent a skin prick test with 15 standard aero-allergens and five food allergens (ALK-Abellò, Hørsholm, Denmark). Oedema of >3 mm was accepted as positive.

### Genotyping

A total of 131 EDTA containing blood samples were collected from patients with asthma and stored at −80°C until nucleic acid purification. Residual EDTA containing blood samples collected for routine biochemistry tests were used as the control group. Nucleic acids were isolated from these samples using a commercial genomic DNA extraction kit (Thermo Scientific, MA, USA).

The Rotor-Gene Q 5plex high resolution melting (HRM) platform and Rotor-Gene 6000 series software v1.7 (Qiagen, Hilden, Germany) were used for real time PCR amplification and HRM analysis. 5x Hot Fire Pol Evagreen HRM mix (Solis BioDyne, Tartu, Estonia) used for PCR amplification and PCR reactions were performed in a 20 μL final volume containing 5 μL of purified genomic DNA. 0.1 mM of primers (Table 1) used for amplification. Amplification was performed 95°C for 10 minutes for initial denaturation followed by 30 cycles at 95°C for 10 seconds, 53°C for 45 seconds and 72°C for 45 seconds. After amplification, denaturation was performed from 72°C to 90°C for HRM analysis. Representative samples of genotypes were confirmed by DNA sequencing for TLR4 polymorphisms and via the restriction fragment length polymorphism (RFLP) method for the CD14 polymorphism. Representative samples were also used controls for further tests (Table 1) [14,17].

**Table 1:** Primers used in this study

**Primer name**	**Sequence**	**References**
TLR_299_F	TGA AGA ATT CCG ATT AGC ATA CTT AGA	[17]
TLR_299_R	CTT TCA ATA GTC ACA CTC ACC AG	In this study
TLR_399_F	TGA GTT TCA AAG GTT GCT GTT CTC	[17]
TLR_399_R	GTT TGA ACT CAT GGT AAT AAC ACC	In this study
CD14-159_F	GCCTCTGACAGTTTATGTAATC	[14]
CD14-159_R	GTGCCAACAGATGAGGTTCAC	[14]

### Statistical analysis

Statistical analyses were performed using the SPSS 16 package software (SPSS Inc., Chicago, IL, USA). Patient demographics and disease characteristics were summarised using descriptive statistics. All numeric data were expressed as the mean ± standard deviation, and non-numeric data as frequencies and percentages. Statistical differences between group means were analysed using Student’s t-test. One- way analysis of variance (ANOVA) was used to compare means among more than two groups, and Tukey’s HSD test was used in the post-hoc analysis as necessary. A chi-square test was used to determine whether allele and genotype frequencies in the asthma and healthy control groups deviated from the Hardy–Weinberg equilibrium. The chi-square test was used for non-numeric data. A p value of <0.05 was accepted as statistically significant.

## Results

Characteristics of the asthma and control groups are shown in Table 2. No significant differences were observed among the CD14 -159, TLR4 299, and TLR4 399 genotype distributions between the asthma and control groups (p > 0.05). The allele and genetic variant frequencies of the two groups are given in Table 3. CD14 C-159 T, TLR4 299 and TLR4 399 genotypes, total IgE levels, eosinophil counts, and FEV1 were evaluated according to severity of disease and atopy status (Tables 4 and 5). To determine factors affecting total IgE levels, we performed a logistic regression analysis using the backward stepwise method for variable selection based on the likelihood ratio. Because the median IgE level in the asthmatic patients was 98 kU/L, this value was used as the threshold for calculations in the logistic regression analysis (Table 6). A significant correlation was found between IgE levels and allergic dermatitis, eosinophil counts, and skin prick test results.

**Table 2:** Characteristics of asthma and control groups

**Characteristics**	**Number of cases (n)**	**Mean (Standart deviation)**	**Range**
Age (Year)	131	36 (12.42)	18–68
Atopic	96	31 (8.72)	18–57
Non-atopic	35	52 (6.57)	42–68
Control	75	43 (7.68)	23–65
Female (Total)	96		
Atopic	63		
Non-atopic	30		
Control	50		
Male (Total)	35		
Atopic	33		
Non-atopic	5		
Control	25		
Duration of asthma (Year)	131	4.52 (5.28)	0–35
FEV1 (%)			
Asthma	131	68.60 (12.67)	37–97
Control	75	95.70 (14.56)	90–115
Total IgE (kU/I)			
Asthma	131	175.77 (220.28)	4–986
Control	75	45.32 (30.25)	5–89
Eosinophil (%)			
Asthma	131	3.39 (2.9)	0–14
Control	75	1.10 (0.5)	0–3
Skin prick test (Asthma)			
Positive	96		
Negative	35		

FEV1: Forced expiratory volume 1.

**Table 3:** Genotype-allele distribution in asthma and control group

**Genotypes/Alleles**	**Asthma patients n (%)**	**Control group n (%)**	**p**
CD14-159			
CC	26 (19.3)	15 (19.4)	0.99 (NS)*
CT	63 (49.2)	36 (49.2)	
TT	42 (31.5)	24 (31.4)	
CC + CT (C allele)	115(43.9)	66 (44)	
TT (T allele)	147(56.1)	84 (56)	
TLR4 299			
AA	122 (93.1)	71 (94.7)	0.77 (NS)*
GA	9 (6.9)	4 (5.3)	
A allele	253 (96.6)	146 (97.3)	
G allele	9 (3.4)	4 (2.7)	
TLR4 399			
CC	120 (91.6)	71 (94.7)	0.58 (NS)*
CT	11 (8.4)	4 (5.3)	
C allele	251 (95.8)	146 (97.3)	
T allele	11 (4.2)	4 (2.7)	

NS: Not significant.

*Chi-square test.

**Table 4:** Genotypic- phenotypic features in all asthma patients with mild, moderate and severe asthma

	**All asthma patients**			**p**	**Atopic**	**Non-atopic**	**p**
	**(Standart deviation)**				**Asthmatics (Standart deviation)**	**Asthmatics (Standart deviation)**	
	1	2	3		(n = 96)	(n = 35)	
	(n = 19)	(n = 78)	(n = 34)				
FEV1 (%)	88	72	51	**<0.001**	71	62	**<0.001**
	(6.7)	(4.2)	(6.2)		(12)	(13)	
Eosinophil (%)	3.25	4.02	3.61	**<0.001**	3.84	2.2	**0.008**
	(3)	(3.35)	(2.5)		(3.2)	(1.3)	
Total IgE (kU/L)	170	240	199	**0.006**	223	47	**<0.001**
	(156)	(255)	(244)		(240)	(37)	
Log IgE median	1.9	2.02	1.7	**0.006**	2.1	1.5	**<0.001**
	(0.5)	(0.6)	(0.6)		(0.5)	(0.4)	
CD14-159							
CC	1	14	5	0.27	20	6	0.62
CT	5	31	7		43	19	
TT (T Allele)	9	21	3		33	10	
CC+CT (CAllele)	6	45	12	0.10	63	25	0.68
TLR4 299							
AA	14	60	14	0.45	88	34	0.44
GA	1	6	1		8	1	
TLR4 399							
CC	14	58	14	0.23	86	34	0.29
CT	1	8	1		10	1	

Comperative Features in Atopic and Non-atopic Asthmatics.

1: Mild asthma; 2: Moderate asthma; 3: Severe asthma. FEV1: Forced expiratory volume 1. Log IgE: Logarithm IgE.

Significant p value is shown with black boldface.

**Table 5:** Genotypic-phenotypic features in atopic and non-atopic patients with mild, moderate and severe asthma

	**Atopic asthmatics**			**p**	**Non-atopic asthmatics**			**p**
	**(Standart deviation)**				**(Standart deviation)**			
	1	2	3		1	2	3	
	(n = 15)	(n = 66)	(n = 15)		(n = 4)	(n = 12)	(n = 19)	
FEV1 (%)	88	72	51	**<0.001**	89	69	52	**0.03**
	(6.7)	(4.2)	(6.2)		(4.4)	(5.1)	(4.9)	
Eosinophil (%)	3.25	4.02	3.61	0.73	1.50	1.93	2.43	0.46
	(3)	(3.35)	(2.5)		(0.6)	(1.2)	(1.4)	
Total IgE (kU/L)	170	240	199	0.63	63	40	43	0.49
	(156)	(255)	(244)		(47)	(33)	(38)	
Log IgE median	2	2.1	2	0.75	1.7	1.4	1.5	0.49
	(0.5)	(0.5)	(0.6)		(0.5)	(0.5)	(0.4)	
CD14-159								
CC	1	14	5	0.14	1	1	4	0.70
CT	5	31	7		2	7	10	
TT (T Allele)	9	21	3		1	4	5	
CC + CT (C Allele)	6	45	12	**0.049**	3	8	14	0.82
TLR4 299								
AA	14	60	14	0.92	4	11	19	0.41
GA	1	6	1		-	1	-	
TLR4 399								
CC	14	58	14	0.70	4	11	19	0.41
CT	1	8	1		**-**	1	-	

1: Mild asthma; 2: Moderate asthma; 3: Severe asthma. FEV1: Forced expiratory volume 1. Log IgE: Logarithm IgE.

Significant p value is shown with black boldface.

**Table 6:** Logistic regression analysis for total IgE level >98 kU/L

		**Multivariate**	
	OR	95% CI	p
Age	−	−	−
Sex	−	−	−
Allergic rhinitis	−	−	−
Allergic dermatitis	8.79	1.81-46.88	**0.007**
Severity of asthma	−	−	−
Eosinophil (%)	1.54	1.23-1.93	**0.0001**
Skin-prick test reactivity	4.90	1.64-14.62	**0.004**
CD14 -159	−	−	−
TLR4 299	−	−	−
TLR4 399	−	−	−

OR: Odds ratio; CI: Confidence intervals. 98 kU/L: Median Total IgE level.

Significant p value is shown with black boldface.

**Figure 1:**
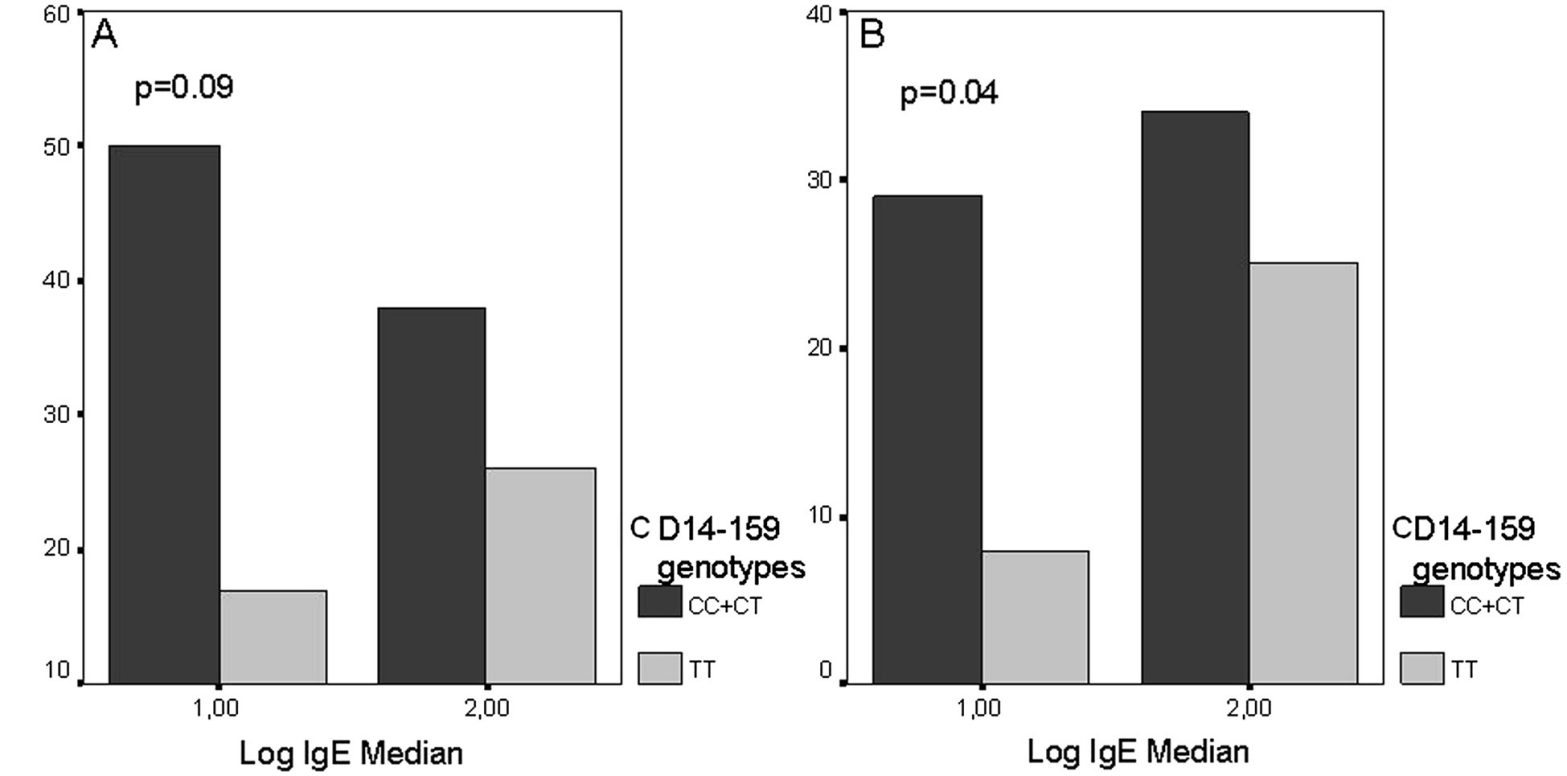
**The association between median log IgE and CC+CT and TT genotypes in all asthma patients and atopic asthma group. A-**The insignificant association between median log IgE (according to 1.99) and CC + CT and TT genotypes in all asthma patients (p = 0.09). **B-**The significant association between median log IgE (according to 1.99) and CC + CT and TT genotypes in atopic asthma group (p = 0.04).

**Figure 2:**
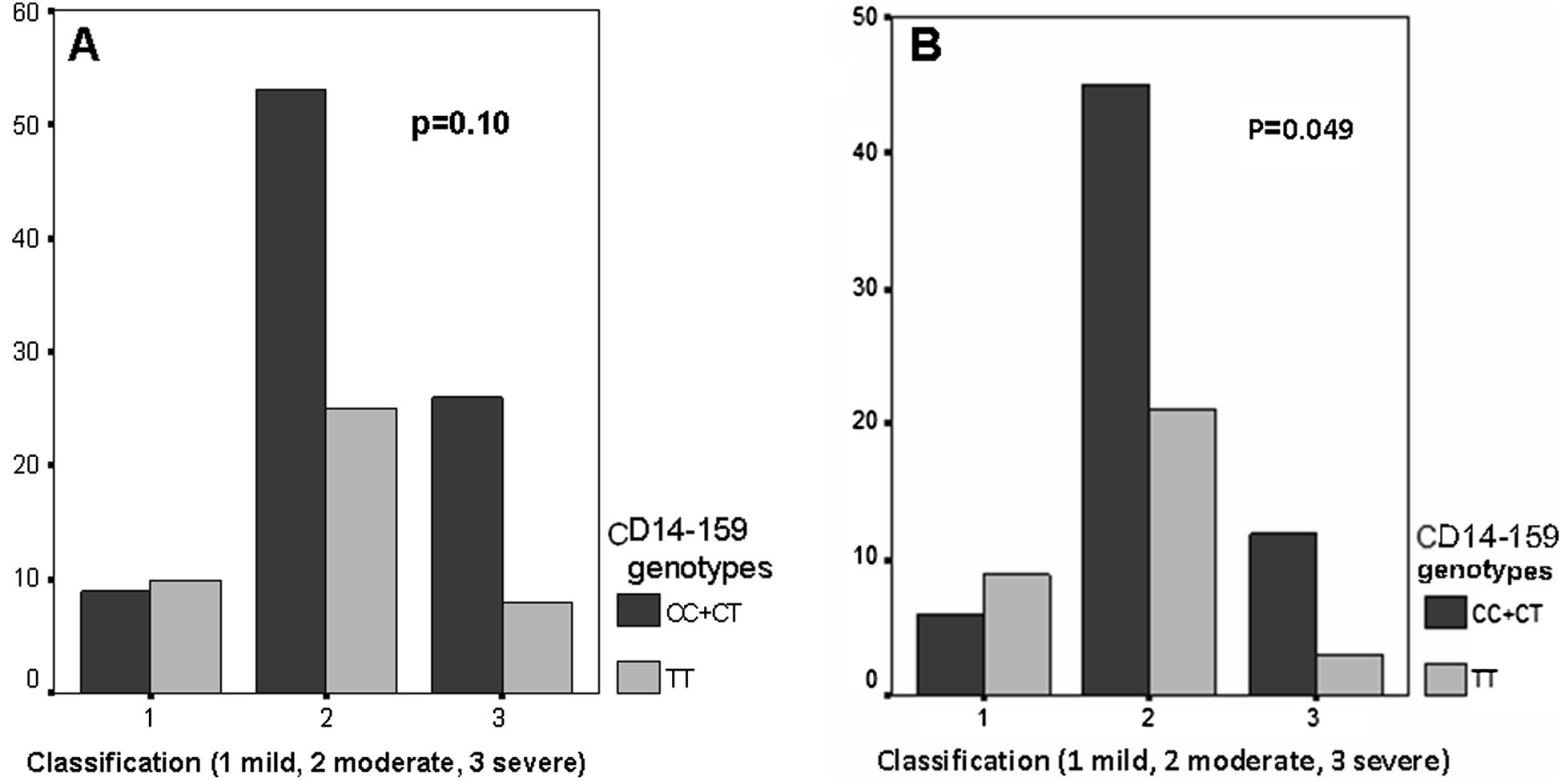
**The association between the severity of asthma and CC+CT and TT genotypes in all asthma patients and atopic asthma group. A-**The insignificant association between the severity of asthma and CC + CT and TT genotypes in all asthma patients (p = 0.10). **B-**The significant association between the severity of asthma and CC + CT and TT genotypes in atopic asthma group (p = 0.049).

Total IgE levels were converted to a normal distribution using logarithmic (log) transformation. No significant difference was observed when individually comparing the median log IgE (according to 1.99) among patients with the CC, CT, TT genotypes of the CD14C-159 T polymorphism (p = 0.08); in the asthma group, presence of the C allele (CC + CT) was more frequent among those with low IgE levels, but no statistically significant difference was detected (p = 0.09, Figure 1A). Patients in the asthma group were classified as either atopic and non-atopic according to the results of the skin prick test and history of atopic rhinitis-dermatitis A significant association was observed between skin prick test positivity and high total IgE levels and eosinophil counts. Moderate asthma was more frequent in the atopic group and severe asthma was found in the non-atopic group; FEV1 was lower in both the atopic and non-atopic groups. In atopic patients, there was a significant association between the median log IgE (according to 1.99) and the presence of the C allele (p = 0.04, Figure 1B). As a result, it was found that the C allele was significantly correlated with low total IgE levels and that the T allele was significantly correlated with high total IgE levels. However, among all asthma patients, no significant association was observed between the severity of asthma and CC + TT and TT (p = 0.10, Figure 2A). In the atopy group, a significant association was also observed between the severity of asthma and CC + TT and TT genotypes (p = 0.049, Figure 2B). Specifically, the C allele was significantly more frequent in the moderate and severe asthma groups.

The TLR4 299 and TLR4 399 genotypes were not significantly associated with asthma phenotype (p > 0.05). Heterozygous TLR4 genotypes (TLR4 A896G and TLR4 C1196T polymorphisms) were more frequent in the moderate asthma group, but this association was not statistically significant. No significant difference was observed between the atopic and non-atopic groups.

## Discussion

The CD14 gene is present in the major susceptibility region (5q31-33) for atopy and asthma [17,18]. Studies investigating the relationship between the CD14 gene and atopy and asthma have revealed conflicting results. First, Baldini et al. [12] described several single nucleotide polymorphisms in the CD14 gene, which is a receptor with high affinity for LPS. The polymorphisms described were found to affect the binding affinity for Sp1, -2, and −3 transcription factors at the GC box in vitro, thus altering the expression of CD14. They also detected a C-T substitution in the promoter region of the gene (the CD14-159 T polymorphism) and higher CD14 levels and low skin test sensitivity in TT homozygotes [12]. The CD14 C-159 T polymorphism involves a C → T nucleotide substitution, located −260 bp from the translation start site and −159 bp from the transcription start site, which alters CD14 promoter activity in vitro by decreasing the affinity of Sp protein binding and thus enhancing transcriptional activity [19]. As a result, the CD14 C-159 T promoter polymorphism was associated with serum CD14 levels [12,20] as well as the phenotypes of patients with allergy [18,21]. In a meta-analysis reviewing studies of Asian population and children, the TT and CT genotypes were correlated with decreased atopic asthma risk compared to CC [15,22]. On the other hand in some studies have reported that the C allele [23] was a risk factor and CC genotype was found to be correlated with positive skin prick test [24]. Another meta-analysis reported that among individuals with TT genotypes exposure to low levels of endotoxins was protective for asthma, but high levels of endotoxin increased the risk of asthma. Conversely, in individuals carrying the C allele exposure to high levels of endotoxin had a protective effect [25]. In another study, increasing endotoxin exposure was associated with a reduced risk of allergic sensitisation but and increased risk of non-atopic wheezing in children with the CC genotype at position −159 of the CD14 gene [26]. In our study the distributions of the CD14 genotypes (CC, CT, TT) did not differ significantly between the asthma and control groups. Unlike the studies mentioned no significant correlation was observed between the CD14 genotype and positive skin test, however the CD14 genotype was correlated with the level of total IgE and severity of disease. Studies investigating the correlation between total IgE levels and the CD14 genotype revealed conflicting results. In one study the CD14-159 genotype was correlated with total IgE levels and this correlation was reported to vary according to varying levels of endotoxin exposure [27]. In individuals with the CC genotype both clinical signs of atopic disease and skin test positivity with high serum IgE levels were encountered more frequently [18,28,29]. Similarly in a study of children in India the CD14 C-159 T CC genotype was strongly associated with atopic asthma and serum IgE [30]. Saçkesen et al. [13] reported that the T allele was correlated with low levels of total IgE in children with atopic asthma. The association between C-159 T genotype and allergic sensitisation depends on the level of exposure to endotoxins: TT homozygotes are protected at low levels of exposure and at risk at high levels [29]. These findings suggest an antagonistic interaction between the environment and C-159 T as a determinant of allergic sensitization: the T allele could be either a protective factor or a risk factor, depending on the degree of exposure to environmental microbial products [29]. In other studies C-159 T alleles were not found to be correlated with asthma and atopy (e.g.allergic rhinitis) in children and adults [31-34]. In a study no correlation between C-159 T and serum IgE levels was found [35]. Unlikely there was no significant association between the medianlog IgE concentration and asthma group (atopic and non-atopic) regarding CD14 genotype distribution in our study, although a significant difference was observed within the atopic asthma group for median log IgE levels in relation to the presence of C and T alleles. In contrast to the low log IgE levels in patients bearing C alleles, patients bearing T alleles showed high IgE levels. This result is important because it contrasts with observations in studies reporting that the C allele was correlated with high IgE levels and T allele with low IgE levels. The previously reported prevalence of the CC + CT genotype compared to TT genotype in asthma patients with higher total IgE levels may have resulted from the level of endotoxin exposure as reported in a meta-analysis conducted by Martinez et al. [25]. In other words dominant C or TT allele homozygosity might have a protective or risk-predictive role according to the varying degrees (low or high) of endotoxin and microbial particle exposure [29]. The correlation of CD14 C-159 T genotype with the eosinophil count in asthma was also investigated. Although two previous studies reported no correlation [18,36], eosinophil counts tended to be higher in the CC genotype than in the TT, [13] and to be more common in patients with moderate and severe asthma [21]. Also in our study the correlation between the CD14 genotype and eosinophil count was not statistically significant. The relationship of the CD14 C-159 T genotype with the severity of asthma was also investigated. In some studies the C allele has been associated with moderate-severe asthma and asthma attacks; whereas the T allele is associated with less severe asthma [37,38]. The CD14 C allele was found to be more common in asthma patients who were allergic to house dust. In addition, exposure of these patients to house dust caused moderate-severe asthma attacks [39]. Similarly our study found that the frequency of the C allele was significantly higher in the moderate and severe asthma group.

Genetic variations in TLR1, TLR2, TLR4, TLR6 and TLR10 have been associated with the development of asthma [40]. The TLR4 gene (gene ID 7099) is located at chromosome 9q33.1 [41]. Polymorphisms of TLR4 genes are reportedly related to a decreased response to LPS in humans. Individuals with the Asp299Gly TLR4 polymorphism were shown to have decreased IL-12 and IL-10 levels produced by mononuclear cells stimulated with LPS and a four-fold higher risk of asthma [42]. In a study conducted by Saçkesen et al. on heterozygous Turkish children the TLR4 polymorphism was reported to be related with mild asthma and may protect against severe asthma [13]. On the other hand, other studies have failed to find a correlation between common TLR4 mutations (especially Asp299Gly) and asthma or atopy [8,41,43,44], the severity of asthma may have been a confounding factor in these analyses [41]. Nevertheless TLR4 polymorphism was more closely related to the moderate and severe atopic asthma group than the mild atopic asthma group For the Asp299Gly polymorphism the Asp allele was associated with mild atopic asthma and the Gly allele with moderate and severe asthma [8]. Our study did not detect a relationship between the TLR4 299 or TLR4 399 genetic variants and atopic conditions or asthma , nor did we observe a significant relationship between these genetic variants and the severity of asthma.

## Conclusions

A statistically significant correlation between CD14 genotypes and total IgE levels and severity of asthma was found in our atopic patients. Total IgE levels were found to be low among patients with the CC + CT genotype, and high among patients with the TT genotype contrary to the results of many other studies, which is therefore an important finding. This may be explained by the exposure of atopic asthma patients to varying endotoxin levels as stated in some study reports. Another important finding was that the C allele is a risk factor for moderate and severe asthma. Further studies will allow for the gathering of more detailed information.

## Abbreviations

TLR: Toll like receptor; LPS: Lipopolysaccharides; PAMP: Pathogen associated molecular patterns; APC: Antigen presenting cell; GINA: Global Initiative for Asthma; log: Logarithm; CC + CT: C allele; TT: T allele; FEV1: Forced expiratory volume 1.

## Competing interests

The authors declare that they have no competing interests.

## Authors’ contributions

FŞ performed study design, sample collection, interpretation of data and the writing manuscript. PY performed the statistical data analysis. AK, MAK, NK and KM carried out the genotyping assays. All authors read and approved the final manuscript.

## Pre-publication history

The pre-publication history for this paper can be accessed here:

http://www.biomedcentral.com/1471-2466/14/20/prepub
